# Estimation of Biomass Burning Influence on Air Pollution around Beijing from an Aerosol Retrieval Model

**DOI:** 10.1155/2014/649648

**Published:** 2014-08-27

**Authors:** Sonoyo Mukai, Masayoshi Yasumoto, Makiko Nakata

**Affiliations:** ^1^Kyoto College of Graduate Studies for Informatics, 7 Tanaka Monzencho, Sakyo, Kyoto 606-8225, Japan; ^2^Kinki University, 3-4-1 Kowakae, Higashi-Osaka, Osaka 577-8502, Japan

## Abstract

We investigate heavy haze episodes (with dense concentrations of atmospheric aerosols) occurring around Beijing in June, when serious air pollution was detected by both satellite and ground measurements. Aerosol retrieval is achieved by radiative transfer simulation in an Earth atmosphere model. We solve the radiative transfer problem in the case of haze episodes by successive order of scattering. We conclude that air pollution around Beijing in June is mainly due to increased emissions of anthropogenic aerosols and that carbonaceous aerosols from agriculture biomass burning in Southeast Asia also contribute to pollution.

## 1. Introduction 

Atmospheric aerosol distributions in East Asia are known to be complicated, owing to both natural factors and human activity. In urban areas, small anthropogenic aerosols dominate because of emissions from diesel vehicles and industrial activity. Aerosol distribution in East Asia is especially known to be heavily affected by increased emissions of sulfuric, nitric, carbonaceous, and other aerosols associated with economic growth [1]. Increased emissions of anthropogenic particles cause concentrations of serious air pollutants. Large cities in eastern and southwestern China in particular have experienced heavy haze episodes (dense concentrations of atmospheric aerosols) over the past thirty years. However, the aerosol properties of these events are still insufficiently understood.

This work develops an efficient algorithm for aerosol retrieval in haze episodes around urban areas in China. Aerosol distribution varies seasonally due to factors such as emissions, photochemical reactions, and wind direction [2, 3]. Furthermore, yellow dust events—some of the most dynamic natural phenomena to produce atmospheric aerosols—can increase particulate matter (PM) concentrations and cause serious atmospheric turbidity [4]. Atmospheric aerosols also influence climate because they play an important role in global environmental change [5] and meteorology [2, 6]. The 5th IPCC report on the global warming problem (https://www.ipcc.ch/report/ar5/wg1/) emphasizes the importance of observing aerosol characteristics and their temporal and spatial variations and points out the warming effect of black carbon aerosols against the cooling effect of other kinds of aerosols.

Our research group has been retrieving aerosol characteristics such as amount, size, composition, and shape based on satellite data [7, 8], ground measurements [9], and numerical model simulations [10]. In this study, we focus on aerosol characteristics around Beijing during a severe air pollution episode in June 2010 using simultaneous space-based MODIS sensor products [11] and ground-based Cimel photometer measurements, analyzed using a standard Aerosol Robotics Network (AERONET) processing system [12–14]. It is natural to consider that incident solar light multiply interacts with atmospheric aerosols due to a dense radiation field in such heavy episodes. The method of successive order of scattering (MSOS), which allows simulation of radiation reflected from an optically semi-infinite atmosphere, has been examined [15]. Here our MSOS code is evaluated from synthetic points of view such as space and ground measurements and model simulations.

The remainder of this paper is organized as follows: Section 2 interprets the motivation of this work on the air pollution episodes detected at the end of June 2010 around Beijing, from ground- and space-based measurements.  Section 3 interprets our aerosol retrieval algorithms as applied to dense aerosol episodes.  Section 4 presents the retrieved results of aerosol properties around Beijing on June 25, 2010, along with numerical model simulations. Finally, Section 5 presents a brief summary of our work and implications of our method.

## 2. Motivation

**Figure 1:**
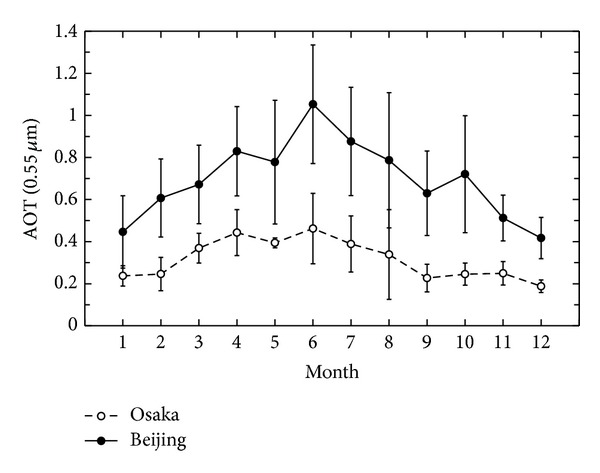
Monthly averaged AOT over the 10 years from 2002 to 2012 at the Beijing and Osaka AERONET sites. Error bars show monthly standard deviations.

Figure 1 presents the monthly averages of aerosol optical thickness (AOT) at a wavelength of 0.55 *μ*m over AERONET sites at Beijing (black circles) and Osaka (white circles) during the ten years from 2002 to 2012. AOT is an important aerosol parameter that can be derived from the transmittance measured by direct sun photometry. The following results are inferred from this figure.

1. AOT values over Beijing are very high, as compared with Osaka.
2. AOT peaks exist in June at both sites. Beijing has a particularly sharp peak in June. The peak in June in Beijing is caused by haze events, which seem to be partly caused by the forest fire and/or field burning in southeastern China.
3. The second peak in April is caused by yellow sand events.

Worldwide NASA/AERONET data are mostly available as ground-based sun-photometric products. They provide the aerosol optical thickness (AOT (*λ*)) at wavelength *λ*. AOT resolution is better than 0.01 at all observation wavelengths, and the obtained data are cloud-screened before aerosol retrieval [16]. Several other aerosol parameters, such as the Ångström exponent *α* defined by (1) [17], size distribution, and refractive index, are derived from the basic data of spectral AOT (*λ*):

(1) $$\begin{array}{c} \alpha =\frac{-\ln\left(\text{AOT}\left(\lambda_{2}\right)/\text{AOT}\left(\lambda_{1}\right)\right)}{\ln\left(\lambda_{2}/\lambda_{1}\right)}. \end{array}$$

Values of *α* are closely related to the aerosol size; small values of *α* indicate large particles and large values indicate small particles. In general, values of *α* from near 0 to 1.0 indicate large particles such as sea salt aerosols and soil dusts, whereas values of 1.0 < *α* indicate particles such as sulfate and those associated with biomass burning [18]. Aerosol episodes are simply defined as periods of high AOT values. However, each aerosol episode has its own characteristics. Some aerosol episodes show the characteristic features of dust storms with high AOT and low *α* values, but detection of high *α* values almost always indicates contamination by small anthropogenic particles.

**Figure 2:**
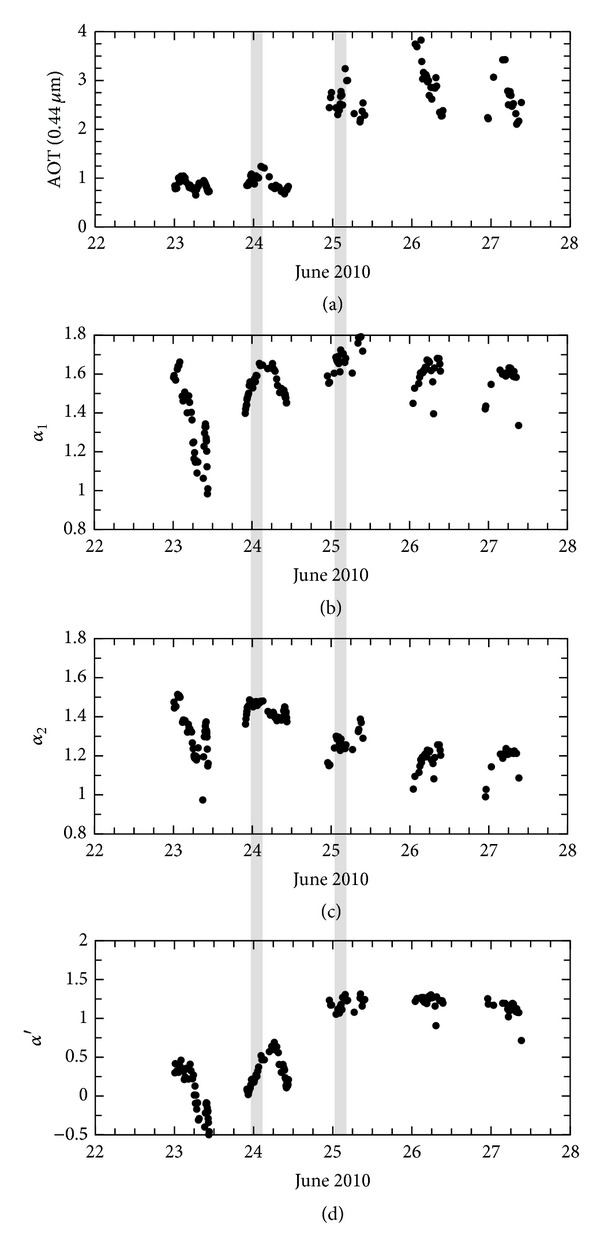
AERONET data (Level 2) at the AERONET Beijing site in June 2010. Figures (a), (b), (c), and (d) represent AOT (0.44 *μ*m), Ångström exponent *α*_1_(*λ*_3_, *λ*_2_), *α*_2_(*λ*_2_, *λ*_1_), derivatives of the Ångström exponent *α*′, where *λ*_1_, *λ*_2_, and *λ*_3_ take values of 0.44, 0.675, and 0.87 *μ*m, respectively.

Figure 2 shows these aerosol optical properties (Level 2) observed at the AERONET Beijing site at the end of June 2010. The upper figure (Figure 2(a)) shows the aerosol optical thickness at a wavelength of 0.44 *μ*m (AOT (0.44 *μ*m)). The middle figures (Figures 2(b) and 2(c)) show Ångström exponent *α*. High values of *α* suggest the dominance of small aerosols. It is clear from (1) that values of *α* depend on the wavelength. Figure 2(d) shows the derivatives of the Ångström exponent (*α*′) [17, 19]:

(2) α′=dαdln⁡⁡λ=−2({ln⁡⁡(τλ3/τλ2)ln⁡⁡(λ3/λ2)−ln⁡⁡(τλ2/τλ1)ln⁡⁡(λ2/λ1)}) ×(ln⁡(λ3λ1))−1.

The value of *α*′ appears to indicate spectral variation of the particle properties. At the very least, the sign of *α*′ appears to signify some particle properties, in that negative and positive values correspond to dry and absorbing particles, respectively. The shaded parts in Figure 2 indicate the appearance of an aerosol episode starting on June 25, as the AOT values suddenly increase in comparison with low values on June 24. Moreover values of *α*′ increase with the AOT values, being low on June 23–24 and high from June 25 to 27. This feature of *α*′ suggests the presence of carbonaceous aerosols during the aerosol episode starting on June 25. These ground measurements suggest that an aerosol episode with small pollutants occurred around Beijing starting on June 25, 2010, and that carbonaceous aerosols played some role in this episode. Certainly, there exist carbonaceous aerosols from local sources such as industry and automobiles. To examine this aerosol episode suspected from ground measurements, we use satellite observations.

**Figure 3:**
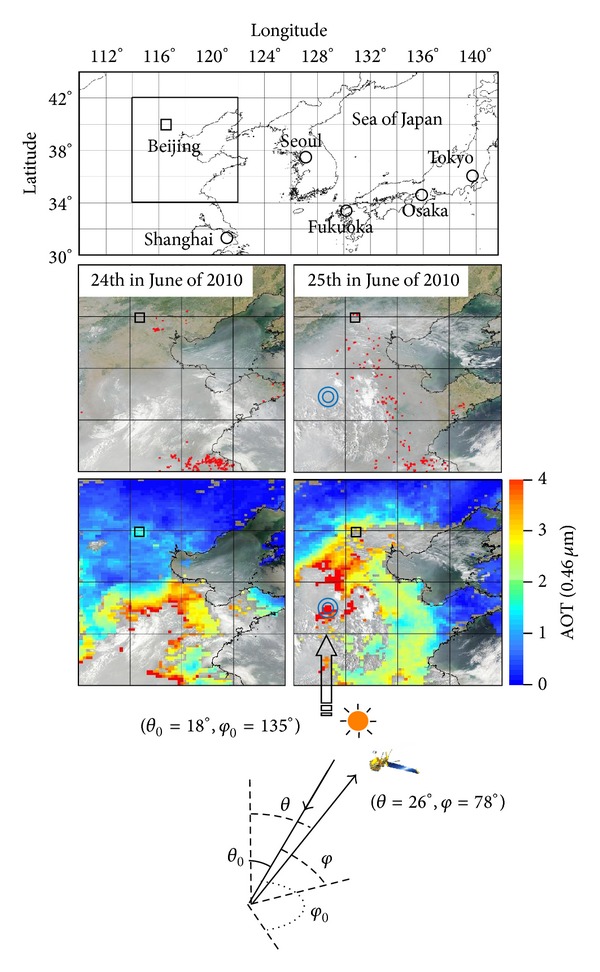
Aqua/MODIS images over Beijing on June 24 and 25, 2010. The top, middle, and bottom figures present a geographical map of East Asia, composite images, and optical thickness of a wavelength at 0.46 *μ*m, respectively (MYD04_L2 Collection 5.1). The □ symbol indicates the position of the AERONET Beijing site and ⊚ denotes the aerosol retrieval target area. The bottom diagram represents the directional information of the target.

Figure 3 shows images of the Beijing area, denoted by the square in the upper map of East Asia, obtained by a MODIS sensor mounted on the Earth-observing satellite Aqua. The middle is a composite image (MYD04_L2 Collection 5.1) of the Beijing area on June 24 and 25, 2010. A square symbol (□) denotes the AERONET Beijing site (39.97 N, 116.38 E). These space-based images coincide with the ground measurements in Figure 2(a), in that the atmosphere over the Beijing AERONET site is clear on June 24 and opaque on June 25. The small red dots in the right lower corner of the MODIS images denote hot spots, which seem to indicate agriculture biomass burnings [20]. They seem to well explain the definite variation of *α*′ from June 24 to 25 in Figure 2(b). It seems that the satellite observation coincides with simultaneous measurements from the ground. A double circle symbol (⊚) denotes the target area for aerosol retrieval. Directional information of the target area such as solar incidence and satellite observation is shown in the bottom diagram.

Assuming values of AOT (0.46 *μ*m) ≥ 4.0 as the criteria for an aerosol episode [15], the area denoted by a double circle symbol (⊚) in Figure 3 clearly shows an episode. This episode also seems to be partly caused by the biomass burnings represented by hot spots, which suggests transportation of dense air parcels from the southeast (where agricultural biomass burning usually occurs during this season) to the northwest.

## 3. Algorithms

The space-borne sensors measure upwelling radiance at the top of the atmosphere (TOA), that is, the reflectance (*R*) from the Earth's atmosphere. It is known that incident solar light multiply interacts with atmospheric particles. The multiple scattering calculations (i.e., radiative transfer problem) take into account Rayleigh scattering by molecules and Mie scattering by aerosols in the atmosphere. In the case of an aerosol episode, we assume an optically semi-infinite atmosphere where radiation incident on the bottom surface is not considered, allowing treatment of only the reflectance* R* for the semi-infinite case. Now* I* is defined to be the specific intensity in the direction of *Ω*, given by *Ω* = (*μ*, *φ*), *dΩ* = *dμ* *dφ*, where *μ* is the cosine of the zenith angle *θ* (so *μ* = cos⁡*θ*) and φ is the azimuth angle. The intensity of the upward radiation (*I*(0, +*Ω*)) at TOA takes the following form:

(3) $$\begin{array}{c} I\left(0,+\Omega \right)=\frac{\mu_{0}}{4}R\left(\Omega ,\Omega_{0}\right)F. \end{array}$$

Here, the function *R* denotes this upwelling radiance and *F* is the incident flux of solar radiation. *I*(0, +*Ω*) is considered to be the case where *πF* enters the top of the atmosphere from the −*Ω*_0_ direction.

In this research, upwelling radiance *R* is efficiently calculated in the optically very thick atmospheric model of aerosol episodes. As mentioned above, the atmosphere of AOT (0.46 *μ*m) > 4 is optically semi-infinite. We propose calculating the reflectance* R* as a sum of the* n*th-order of reflection function R*(*n*) [21, 22]:

(4) $$\begin{array}{c} R\left(\Omega ,\Omega_{0}\right)=\overset{\infty }{\underset{n=1}{\sum }}\omega^{n}R^{*}\left(n:\Omega ,\Omega_{0}\right), \end{array}$$

where *n* is the number of times of scattering, and *ω* represents single-scattering albedo. The* n*th-order reflection function R*(*n* : *Ω*, *Ω*_0_) describes radiation emerging at the TOA after scattering *n* times within the atmosphere. After a large number *n* of interactions R*(*n* : *Ω*, *Ω*_0_) takes the asymptotic form

(5) $$\begin{array}{c} R^{*}\left(n:\Omega ,\Omega_{0}\right)=A\left(\Omega ,\Omega_{0}\right)n^{-3/2}\exp\left[\frac{-d\left(\Omega ,\Omega_{0}\right)}{n}\right], \end{array}$$

where functions *A* and *d* are derived under consideration of stochastic process [23]. Then (4) is rewritten for *n* > n* ≫ 1 as

(6) R(Ω,Ω0)=∑n=1n=n∗ωnR∗(n:Ω,Ω0)+A(Ω,Ω0) ×∑n=n∗+1∞ωnn−3/2exp⁡[−d(Ω,Ω0)n].

Furthermore the second term in the right hand side of (6) is approximated by using error function:

(7) R(Ω,Ω0)=∑n=1n=n∗ωnR(n:Ω,Ω0)+2A(Ω,Ω0)n∗−1/2 ×[e−an∗−(πan∗)1/2erfc⁡(an∗)] −23A(Ω,Ω0)d(Ω,Ω0)n3/2 ×[(1−2an∗)e−an∗+2π(an∗)3/2erfc⁡(an∗)],

where a variable *a* = −ln⁡*ω* and function (erfc) represents the error function. This technique is named the method of successive order of scattering (MSOS), and we call (7) an asymptotic form of MSOS, as opposed to the standard MSOS of (4). Successive scattering is fundamentally related to the probabilistic approach to the theory of radiative transfer. Many papers have been written in this field and much progress has been made, to the extent that a full review exceeds the scope of this study. To interested readers we recommend our recent work [24].

At the first step of radiative transfer, single scattering behavior of the aerosol model as albedo (*ω*) and the phase function *P*(*Ω*, *Ω*_0_) should be determined. The accumulated AERONET data are used to propose the automatic classification of aerosol observations into six categories (DD: desert dust, BB: biomass burning, RU: rural, CP: continental pollution, PM: polluted marine, and DP: dirty pollution) according to their properties at global scales [25, 26]. The data also confirm the consistency and robustness of the method through a cross-validation check. Classification with respect to Asian aerosols is also available using the measurements at Asian-AERONET sites as (Ct-1,-2,-3,-4,-5,-6) [27]. The size distributions of these aerosol types have two modes for small (f: fine) and large (c: coarse) particles, and a bimodal log-normal distribution is assumed as

(8) dVdln⁡⁡r=Vf2πln⁡⁡rfexp⁡[−(ln⁡⁡r−ln⁡⁡rf)22ln⁡2⁡σf] +Vc2πln⁡⁡σcexp⁡[−(ln⁡⁡r−ln⁡⁡rc)22ln⁡2⁡σc].

**Figure 4:**
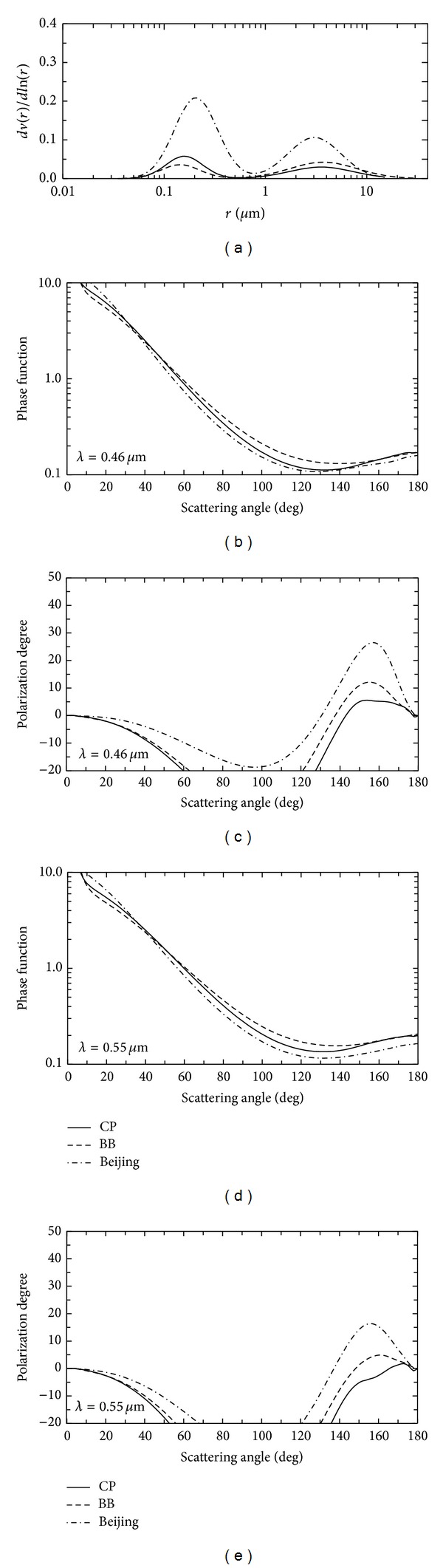
The aerosol model. (a) Size distribution; (b) phase function at *λ* = 0.46 *μ*m; (c) polarization degree at *λ* = 0.46 *μ*m; (d) and (e) are the same as (b) and (c), respectively, but *λ* = 0.55 *μ*m. The solid, dashed, and dot-dashed curves represent continental pollution (CP), biomass burning (BB), and Beijing-type aerosols, respectively.

Here, the left term is the volume particle size distribution (0.05 ≤ *r* ≤ 15 *μ*m). Parameters *V*_*f*_, *r*_*f*_, and *σ*_*f*_ are, respectively, the volume concentration, mode radius, and standard deviation of fine mode particles. The corresponding parameters for coarse mode particles are *V*_*c*_, *r*_*c*_, and *σ*_*c*_. Some of these are shown in Figure 4(a), which also gives the complex refractive index of each type. The fine particle fraction (ff) defined as ff = *V*_*f*_/(*V*_*f*_ + *V*_*c*_) in (8) is available for radiation simulation. Using these AERONET products on clustering work suggests that the aerosol types treated in heavy air pollution roughly correspond to two classes, CP and BB of global classification.

## 4. Results and Discussion

**Figure 5:**
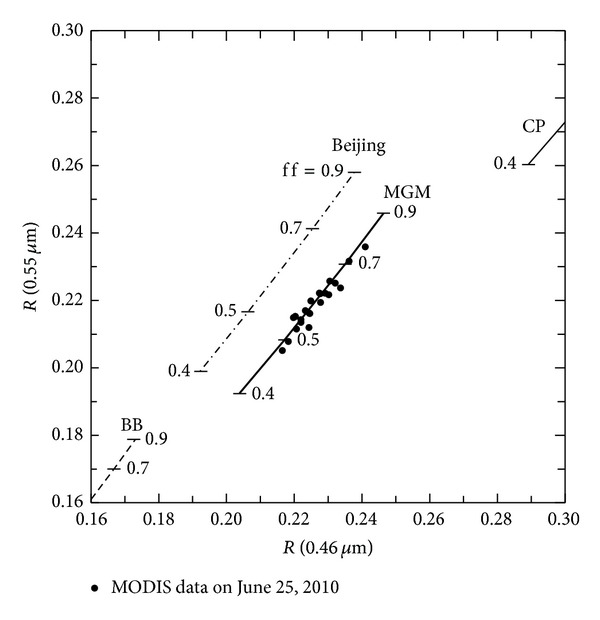
Simulated values of the reflectance* R* for aerosol models of BB (dashed curve), CP (solid curve), Beijing (dot-dashed curve), and MGM (thick solid curve) in a two-channel diagram at wavelengths of 0.46 *μ*m and 0.55 *μ*m. The black filled circles denote MODIS data on June 25, 2010. The variable ff indicates fine particle fraction for size distribution (refer to (8)).

Here we examine aerosol retrieval Aqua/MODIS data from June 25, 2010, from the area indicated by double circles (⊚) in Figure 3. The black filled circles in Figure 5 represent these MODIS data. The solid curves in Figure 5 show the simulated values of the reflectance* R* with MSOS in a two-channel diagram with wavelengths of 0.46 *μ*m and 0.55 *μ*m. In the present simulations, the atmosphere consists of the above-mentioned aerosol types (CP and BB), an aerosol model obtained at Beijing AERONET site (Beijing), and an aerosol type (MGM) optimized to the MODIS observation. The MGM has a complex refractive index calculated from Maxwell Garnet mixing rule [28, 29],

(9) $$\begin{array}{c} \varepsilon =\varepsilon_{m}\frac{\left(\varepsilon_{j}+2\varepsilon_{m}\right)+2f_{j}\left(\varepsilon_{j}-\varepsilon_{m}\right)}{\left(\varepsilon_{j}+2\varepsilon_{m}\right)-f_{j}\left(\varepsilon_{j}-\varepsilon_{m}\right)}, \end{array}$$

where *ε* denotes electricity, subscripts *m* and *j*, respectively, represent matrix and inclusion, and *f*_*j*_ is the volume fraction of the inclusion. The matrix is assumed to be the CP type and inclusion the BB type. The aerosol parameters in our present calculations are size distribution functions, which are represented by the fine particle fraction and refractive indices provided in Table 1. Table 1 also presents the values of complex refractive indices, *m*(*λ*) = *n*(*λ*) − *k*(*λ*)*i*, adopted for aerosol properties for the numerical simulations of* R* shown in Figure 5. The single scattering phase function and polarization degree for aerosol types available for present calculations are from Figure 4.

Figure 5 shows that the MGM-type aerosol is consistent with the MODIS data. Furthermore, we can rank the retrieved aerosols according to their fit to the MODIS data on June 25, 2010, around Beijing, from best to worst:

1. MGM type.
2. Beijing type.
3. CP-type and BB-type global clustering.

The reflectance values obtained using the CP-type are too high and the BB-type aerosol are too low compared to the MODIS data. We thus conclude that the local Beijing aerosol type is a much better fit to interpret the local observations. These results seem reasonable, suggesting that detailed measurements at both spatial and temporal scales are necessary for precise analysis of aerosols. The best candidate MGM-type aerosol looks a modified version of the Beijing type with slightly stronger absorbing particles and suggests the inclusion of carbonaceous aerosols [30]. Transportation of biomass burning aerosols [20, 31] is observed in the satellite images in Figure 3 on June 24 and 25, where carbonaceous aerosols appear to be transported from the southwest toward Beijing.

**Figure 6:**
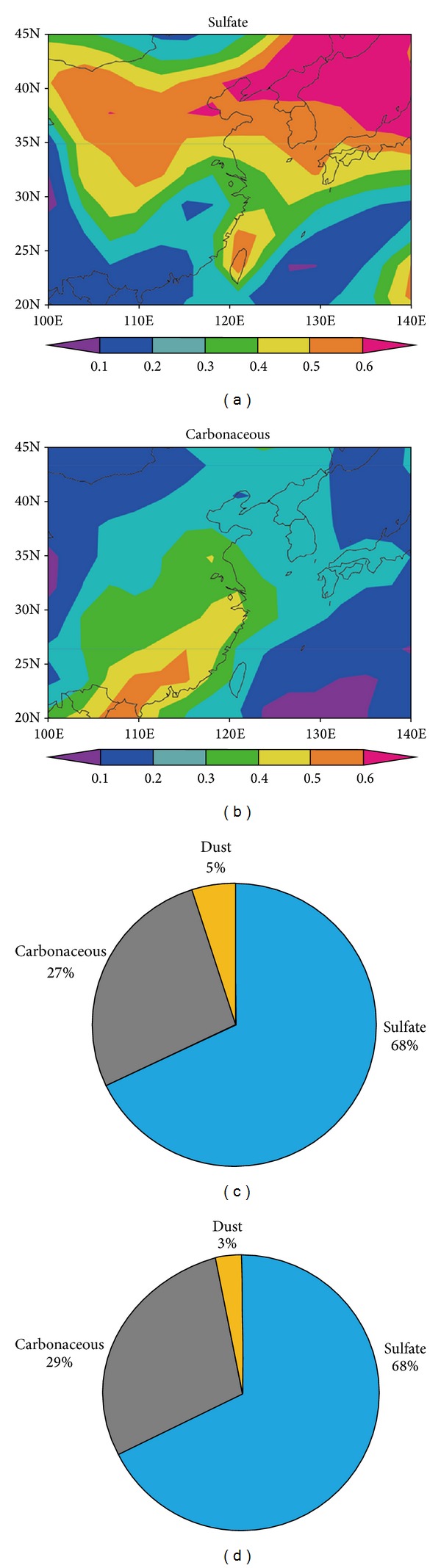
Results of numerical model simulations with SPRINTARS. (a) Distribution of ratios of simulated sulfate AOT components relative to the total AOT at a wavelength of 0.55 *μ*m. (b) The same as (a) but for carbonaceous components. (c) AOT contribution (%) of each component relative to total particles at the Beijing AERONET site (39.97° N, 116.38° E). (d) The same as (c), but at the reference area (37.06° N, 115.35° E) (the ⊚ symbol in Figure 3).

Figure 6 shows the numerically simulated distribution of each AOT component relative to the total AOT in East Asia on June 25, 2010. We use the three-dimensional aerosol-transport-radiation model SPRINTARS [32] to model carbonaceous, sulfate, dust, and sea salt aerosols. Figures 6(a) and 6(b) represent the ratio of AOT (0.55 *μ*m) of sulfate and carbonaceous components to the total AOT, respectively.  Figure 6(a) shows that sulfate dominates over East Asia, and Figure 6(b) shows that the carbonaceous component is concentrated in the south. Figures 6(c) and 6(d), respectively, show the contribution of the aerosol components to the simulated AOT for the Beijing AERONET site (39.97 N, 116.38 E) and MODIS reference area (37.06 N, 115.35 E) for aerosol retrieval shown by the ⊚ symbol in Figure 3.

The sulfate component is dominant in both graphs in Figures 6(c) and 6(d), consistent with Figure 6(a). As for the carbonaceous component, the value in the reference area is slightly larger than at the Beijing site. This simulation result supports the hypothesis that the MGM aerosol type, which is the best candidate to interpret MODIS data in Figure 5, is the internal mixing of biomass burning inclusions into the CP-type aerosol (refer to Table 1) and seems to suggest that some of the carbonaceous aerosols are transported from the southeast toward Beijing. Of course, carbonaceous aerosols emanate from local industries or automobiles. However, only in the case of local emissions, the emission volume of particles in urban cities such as Beijing is higher than those in remote rural areas such as the reference target shown for aerosol retrieval in Figure 5.

## 5. Summary and Future Aspects

We focused on aerosol characteristics during severe aerosol episodes detected by both satellite and ground measurements in East Asia. We showed that dense aerosol episodes can be well simulated using a semi-infinite radiative transfer model composed of the proposed aerosol types, which are compiled from accumulated measurements provided by the worldwide aerosol monitoring network (NASA/AERONET). In addition, we applied an efficient procedure for solving the radiative transfer problem for semi-infinite media called the method of successive order of scattering for Aqua/MODIS data around Beijing.

We suggest that polarization information is useful in retrieving aerosol characteristics, because, as shown in Figure 4, polarization degree is more sensitive to aerosol type than radiance alone, especially in the case of backward-direction observations as shown in the bottom diagram in Figure 3. The GCOMC-1 satellite will be launched in the early winter of 2017 by the Japanese Space Agency (JAXA), and a second-generation global imager (SGLI) will be mounted on the GCOM mission. The SGLI involves 18 spectral channels to measure the Earth's reflectance from near ultraviolet to near infrared wavelengths and thermal emissions in the infrared region. The SGLI has two outstanding features with aerosol observations. The first is polarization measurements at near-infrared wavelengths of 0.67 and 0.87 *μ*m. Specifically the stokes parameters (*I*,* Q*, and* U*) are obtained based on the measurements of three linear polarizer angles, as in the POLDER instrument system [33]. A wavelength of 0.38 *μ*m involving SGLI is the second interesting function for aerosol retrieval. The near-UV channel greatly helps us to obtain absorbing particles such as BBA (biomass burning aerosols), in which the imaginary part of the complex refractive index is high in the near-ultraviolet wavelength. This fact is unique characteristics of BBA from other kinds of aerosols. And hence, in the analysis of TOMS (total ozone mapping spectrometer) data, absorption aerosol index (AAI) at 0.38 *μ*m is used for the detection of BBA [34]. This fact is examined now by using GOSAT-1/CAI band 1 (0.38 *μ*m) measurements.

We conclude that air pollution around Beijing is mainly due to increased emissions of anthropogenic aerosols associated with economic growth and to the complicated behavior of natural dust. In June, the high AOT values could be caused by photochemical activity, air mass transportation, high precipitation, and atmospheric stability that could enhance the residence time of particulate matter and increase the levels of particles in the atmosphere. This phenomenon is also found in Osaka in this season (see Figure 1). However, in Beijing carbonaceous aerosols from agriculture biomass burning in Southeast Asia also contribute to the pollution. In that case, high-concentration soot was carried to Beijing from the source area. It is highly likely that large-scale aerosol episodes will continue to occur around Beijing. There are many potential applications for the kind of radiation simulation by MSOS introduced here, because at present the final products of space-based observations can be cut off by an optically thick atmosphere. Air quality is worse in big cities than in remote areas; therefore high-resolution measurements of atmospheric aerosols at spatial, temporal, and spectral scales are needed, especially in urban areas. Polarization observations are also desired.

## Figures and Tables

**Table 1:** Refractive indices for aerosol types in Figure 5.

Aerosol type	Refractive index			
	0.46 (*μ*m)		0.55 (*μ*m)	
	*n*	*k*	*n*	*k*
Continental pollution (CP)	1.420	0.007	1.410	0.007
Biomass burning (BB)	1.510	0.025	1.520	0.025
Beijing (June 25, 2010)	1.459	0.013	1.466	0.012
MGM (*f*_*j*_ = 0.37)	1.453	0.014	1.450	0.014
